# Personality work mediation of space-time relations in extreme situation during COVID-19 lockdown

**DOI:** 10.1192/j.eurpsy.2023.1700

**Published:** 2023-07-19

**Authors:** O. G. Kvasova, M. S. Magomed-Eminov, K. A. Karacheva, I. Prikhodko

**Affiliations:** DEPARTMENT OF PSYCHOLOGY, MOSCOW STATE UNIVERSITY, Moscow, Russian Federation

## Abstract

**Introduction:**

This work presents spatial-temporal relations of time interval estimation in extreme situation. In preliminary analysis of COVID-19 literature and our research (Magomed-Eminov et al, 2021) we identified attributes which people used to describe their experience during lockdown. The categories: limitation of space, freedom, deprivation of action, movement, immobility; negative emotions, disorganization, loss of social contacts, lack of control; avoidance; passive position; victimization - were grouped in factor “restriction of opportunities”.

**Objectives:**

To show experimentally that spatial-temporal relations depend on what meaning a person gives to extreme or non-extreme situation, how she perceives it - hence what inner mental work fulfills in order to find resources to overcome illness, distress, adversity.

**Methods:**

Experimental modelling of extreme situation close to lock-down; content analysis.

**Results:**

In our research-model estimation of short time interval duration in case of self-determination in movement and space and no external limitations of time was almost accurate. In case of limited space and restricted instruction – interval was perceived 2 or 3 times longer than real time. The categories in stories of subjects put in extreme situation instructed to move in one direction in restricted square space opposed to subjects instructed to move freely and in wider space, met the empirical criteria for restriction of opportunities (extreme model) and were categorized in five clusters according to content analysis of self-reports. In brackets we give the features of non-extreme situation (with free instructions to move) characterized by opposite tendencies: a) limitation – featured by boundaries, clamps, tightness (vs freedom); b) negativity of situation perception – by refusal of action, destruction, disorganization, negative emotions, loss (vs positivity); c) static position - by immobility, stiffness, restraint (vs ecstasy, flight, freedom, self-expression); d) avoidance, tendency to escape (vs involvement); e) passive observation and staying in situation (vs active action). The non-extreme features proved to be significantly higher (p<,000) in subjects who received the instruction to move freely in space - opposed to subjects who moved in certain limited space (square).

**Conclusions:**

We revised space-time relations model (D. DeLong; D. Bobko) which demonstrated the tendency for changes of time interval perception in dependence of spatial characteristics. We show another correlation dependent on meaning of situation for individual – extreme or situation of freedom and give interpretation in terms of personality work with negative experience (M. Magomed-Eminov). Research contributes to conception of personality work with one’s own experience in construction of temporal identity, positive outcomes of adversity and meaning mediation while action in extreme situation of lockdown type.

**Disclosure of Interest:**

None Declared

